# Elevated serum level of pancreatic stone protein/regenerating protein (PSP/reg) is observed in diabetic kidney disease

**DOI:** 10.18632/oncotarget.16369

**Published:** 2017-03-18

**Authors:** Ling Li, Dongyu Jia, Rolf Graf, Jiayue Yang

**Affiliations:** ^1^ Department of Endocrinology, Zhongda Hospital, Institute of Diabetes, Medical School, Southeast University, Dingjiaqiao, Nanjing, PR China; ^2^ Women's Cancer Program, Cedars-Sinai Medical Center, Beverly Boulevard, Los Angeles, CA, USA; ^3^ Department of Visceral and Transplantation Surgery, University Hospital of Zurich, Rämistrasse, Zurich, Switzerland; ^4^ Department of Internal Medicine, Division of Diabetes, Metabolism and Endocrinology, Nanjing Medical University Affiliated Wuxi People's Hospital, Wuxi, PR China

**Keywords:** pancreatic stone protein, type 2 diabetes mellitus, diabetic kidney disease, inflammation, serum parameters, Pathology Section

## Abstract

Diabetic kidney disease (DKD) is a major complication of diabetes, and serves as an important cause of end-stage renal disease (ESRD). The role of chronic inflammation in DKD is becoming widely accepted. Pancreatic stone protein/regenerating protein (PSP/reg) is a secretory protein, which is elevated in blood during infected conditions and organ failure. The aim of this study was to investigate the relationship between serum PSP/reg and DKD in patients with type 2 diabetes (T2DM). A total of 120 subjects which includes newly diagnosed T2DM patients, diabetes patients without DKD, DKD patients, as well as healthy controls were enrolled in this study. Serum PSP/reg levels were significantly higher in DKD subjects compared with those of healthy controls (*p* < 0.001), newly diagnosed T2DM (*p* < 0.001) and diabetes patients without DKD (*p* < 0.001). PSP/reg levels correlated positively with glycated hemoglobin (HbA1c) (*p* < 0.001) and serum creatinine (*p* < 0.001). Meanwhile, serum PSP level was negatively correlated with estimated glomerular filtration rate (eGFR) (*p* < 0.001). The area under the curve (AUC) for presence of DKD was 0.854. In conclusion: PSP/reg levels are significantly up-regulated in DKD patients and might be related to renal injury. A follow-up study with a large cohort is needed.

## INTRODUCTION

Diabetes mellitus is one of the most common metabolic disorders in the world. The prevalence of diabetes mellitus was estimated at 382 million in 2013, and is expected to reach 592 million by 2035, due to a dramatic increase in incidence [1]. As an important cause of disability, diabetes mellitus leads to various macrovascular and microvascular chronic complications. Among these complications, Diabetic kidney disease (DKD) is one of the major microvascular complications and is independently correlated with an increased risk of developing end stage renal failure (ESRD). ESRD is a heterogeneous disease with damage of nearly every aspect of the renal structure and function [2–4]. A number of factors have been proposed to cause pathological changes in the kidney of diabetic patients, such as the interactions between hyperglycemia-induced hemodynamics and metabolic changes [5–7], as well as the accumulation of extracellular matrix (ECM) proteins in the glomerular and tubular compartments. However, the exact pathogenesis leading to DKD is not fully elucidated. Recently, accumulated evidence have demonstrated a significant role of chronic low-grade inflammation, termed as ‘microinflammation’ in both the development and progression of DKD [8–11].

Pancreatic stone protein/regenerating protein (PSP/reg) was originally identified in pancreatic calcified concrements, as a 14 kDa insoluble polypeptide belonging to the family of lectin binding proteins [12]. Its 16 kDa precursor has been observed mainly in the pancreas and is transiently elevated in acute and chronic pancreatitis [13]. Under healthy conditions PSP/reg is expressed at low levels in the pancreas, with normal serum levels between 10-15 ng/ml. Upon focal or systemic extra-pancreatic inflammation, PSP/reg is strongly increased [14]. PSP/reg is elevated in various conditions, e.g. sepsis [15], ventilator associated pneumonia (VAP) [16], chronic obstructive pulmonary disease (COPD) exacerbation [17], and type 1 diabetes mellitus (T1DM) [18]. These observations indicated that PSP/reg might respond to infectious conditions and organ failure. Our previous study also found that levels of PSP/reg were up-regulated in type 2 diabetes mellitus (T2DM) and diabetic chronic complications [19].

Understanding the concept that diabetic microangiopathy is associated with inflammation and an important factor in the development of DKD, we evaluated whether serum PSP/reg levels might be associated with DKD and partly predict the future risk. Therefore, we conducted this study to investigate the association between PSP/reg and DKD in T2DM patients.

## RESULTS

### Participants and clinical analysis at baseline

A total of 120 subjects (65 males, 55 females; age range 30-75 years) were enrolled in the study, and were divided into four study populations: healthy control, newly diagnosed T2DM patients, T2DM patients without DKD, and DKD patients. The main clinical and biochemical characteristics of these four study populations are summarized in Table 1.

**Table 1:** Clinical characteristics of subjects

Group	Healthy control	Onset	T2DM without DKD	DKD
**Age (years)**	56.50 [53.75-58.50]	60.00 [56.75-65.00]	63.50 [56.50-67.40]	57.50[54.35-61.20]
**Gender male (%)**	17(56.67)	14 (46.7)	18 (60.0)	16 (53.33)
**Duration (months)**	—	4.93 ±3.18	101.90± 52.87	114.97 ± 53.60
**Smoking (%)**	5 (16.67)	10 (33.33)	7 (23.33)	4 (13.33)
**BMI (kg/cm^2^)**	22.21±2.07	25.51 ± 3.44	24.36 ± 3.35	25.30 ± 3.58
**Systolic BP (mmHg)**	112.57 ± 8.65	118.20± 9.07	131.27 ± 16.26*	136.97 ± 14.60*
**Diastolic BP (mmHg)**	72.00 ± 7.85	73.80 ± 7.12	76.57 ± 9.22	82.83 ± 7.72
**TC (mmol/L)**	4.13 ± 0.75	4.53 ± 0.80	4.80 ± 1.45	4.99 ± 1.40*
**TG(mmol/L)**	1.15 ± 0.26	1.58 ± 0.43*	1.28 ± 0.26	1.98 ± 1.36*
**HDL-cholesterol (mmol/l)**	1.31 ± 0.32	1.12± 0.29	1.15 ± 0.25	1.11 ± 0.21*
**LDL-cholesterol (mmol/l)**	2.23 ±0.43	2.73 ± 0.54	2.89 ± 0.90	2.96 ± 0.99
**OGTT FPG (mmol/l)**	5.15 [4.90–5.30]	7.27 [6.74–8.23]*	5.55 [4.77–7.24]*	7.33 [5.86–8.14]*
**OGTT 2hPG (mmol/l)**	6.05 [5.88–6.30]	11.13 [8.89–15.27]*	10.10 [7.08–12.18]*	11.05 [8.73–14.23]*
**HbA1c (%)**	5.20 [5.08–5.33]	7.55 [6.30–8.53]*	7.05 [6.18–8.23]*	8.25 [7.58–9.10]*
**Creatinine (μmol/L)**	65.27 ± 8.15	62.12 ± 4.59	67.93 ± 10.47	104.77 ± 25.90*
**BUN (mmol/L)**	5.50 ± 0.73	4.55 ± 0.62	4.90 ±1.30	6.18 ± 1.46*
**UA (mg/24h)**	11.90 [4.75-17.26]	15.30 [11.52-22.75]	23.10 [14.98-26.30]	65.10 [31.44-150.0]*
**eGFR (ml/min/1.73 m^2^)**	105.20 ± 11.34	106.21 ± 12.99	100.73 ± 10.86	63.53 ± 19.10*
**CCr (ml/min)**	97.02 ± 17.58	103.64 ± 16.77	98.57 ± 20.39	60.72 ± 22.79*
**Human PSP (ng/ml)**	14.16 ± 4.04	18.74 ± 3.84*	25.84 ± 10.49*	50.32± 20.21*

Clinical characteristics of different groups of subjects. Data are expressed as mean ± SD or as percentages for normal distribution. Non-normally distributed values are presented as median (IQR). BMI, body mass index; Systolic BP, systolic blood pressure; Diastolic BP, diastolic blood pressure; TC, total cholesterol; TG, Triglyceride; HDL, high-density lipoprotein; LDL, low-density lipoprotein; FPG, Fasting plasma glucose; 2hPG, 2h plasma glucose; HbA1c, hemoglobin A1c;BUN, blood urea nitrogen; UA, urinary albumin; eGFR, estimated glomerular filtrations rate; CCr, Creatinine Clearance Rate.*p < 0.05 compared with healthy control.

### Significantly elevated PSP/reg levels were detected in DKD patients

**Figure 1:**
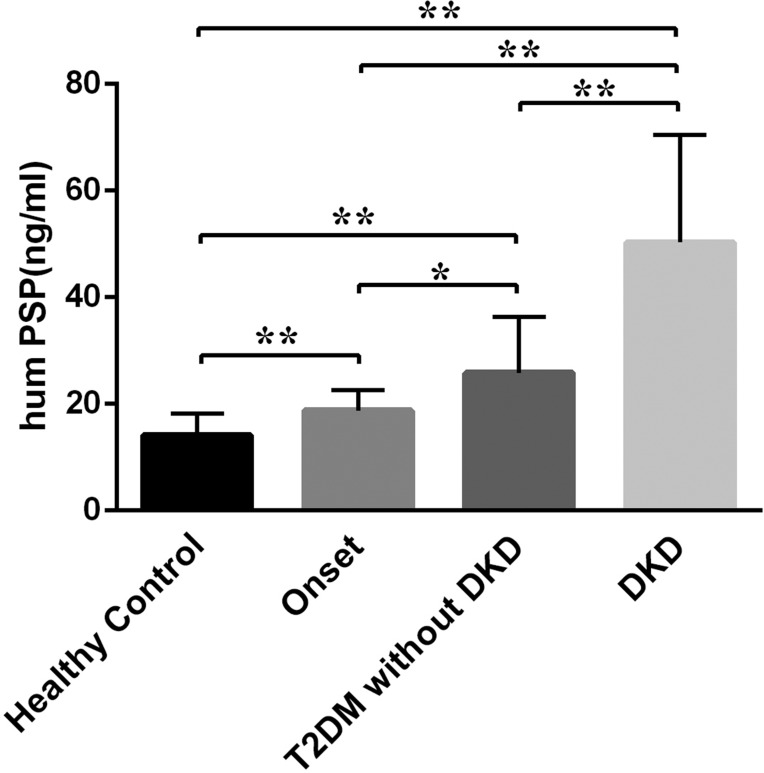
Serum levels of PSP/reg in different groups of the study population PSP/reg serum levels increased significantly in DKD patients (50.32 ng/ml ± 20.21) as compared to healthy controls, newly diagnosed (18.74 ng/ml ± 3.84, *p* < 0.001) and T2DM without DKD patients (25.84 ng/ml ± 10.49, *p* < 0.001). Onset, newly diagnosed T2DM; data are presented as mean ± SD, *p < 0.05, **p < 0.001.

We measured fasting serum PSP/reg levels in all participants using an enzyme-linked immune sorbent assay (ELISA) [15]. Serum levels of PSP/reg from control subjects and diabetic patients are shown in Figure 1. PSP/reg was elevated in T2DM patients and were significantly different as compared to the healthy controls. Notably, we found that PSP/reg levels were remarkably higher in the DKD group (50.32 ng/ml ± 20.21) compared with those in healthy controls (14.16 ng/ml ± 4.04, *p* < 0.001), newly diagnosed (18.74 ng/ml ± 3.84, *p* < 0.001) and T2DM without DKD patients (25.84 ng/ml ± 10.49, *p* < 0.001).

### Serum PSP/reg levels correlated with HbA1c, eGFR, serum creatinine, urinary albumin, and blood pressure

**Figure 2:**
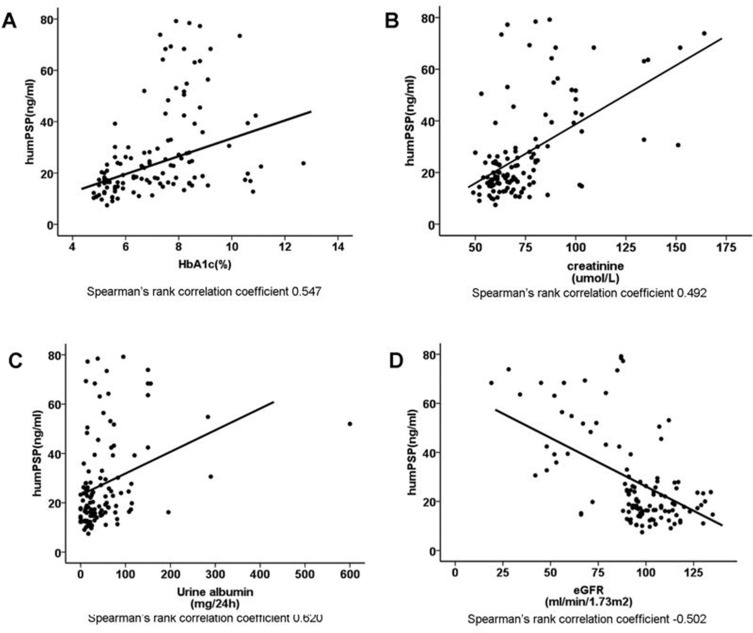
Correlation analysis of PSP/reg in T2DM patients **A**. Correlation of PSP/reg with HbA1c (Spearman *r* = 0.547, *p* < 0.001). **B**. Correlation of PSP/reg with serum creatinine (spearman *r* = 0.492, *p* < 0.001). **C**. Correlation of PSP/reg with UA (Spearman *r* = 0.620, *p* < 0.001). **D**. Correlation of PSP/reg with eGFR (Spearman *r* = -0.502, *p* < 0.001). Lines represent the trend calculated by linear regression.

A significant correlation between PSP/reg level and glycated hemoglobin (HbA1c, Spearman's rank correlation coefficient 0.547, *p* < 0.001, Figure 2A) in all the participants was noted. Simple correlations between PSP/reg and the systolic blood pressure (Spearman's rank correlation coefficient 0.479, *p* < 0.001, Supplementary Figure S1A) and diastolic blood pressure (Spearman's rank correlation coefficient 0.469, *p* < 0.001, Supplementary Figure S1B) were also found. Meanwhile, Serum PSP/reg levels prominently correlated with serum creatinine (Spearman's rank correlation coefficient 0.492, *p* < 0.001, Figure 2B), urinary albumin (UA, Spearman's rank correlation coefficient 0.620, *p* < 0.001, Figure 2C) and negatively correlated with estimated glomerular filtration rate (eGFR, Spearman's rank correlation coefficient -0.502, *p* < 0.001, Figure 2D) and creatinine clearance rate (CCr, Spearman's rank correlation coefficient -0.301, *p* = 0.001). After adjusting for age, sex, BMI, diabetes duration and blood pressure, significant correlations among PSP/reg and HbA1c (*p* = 0.014), eGFR (*p* < 0.001), creatinine (*p* < 0.001), UA (*p* < 0.001), CCr (*p* < 0.001) were still found.

### Relationship between PSP/reg and DKD

**Figure 3:**
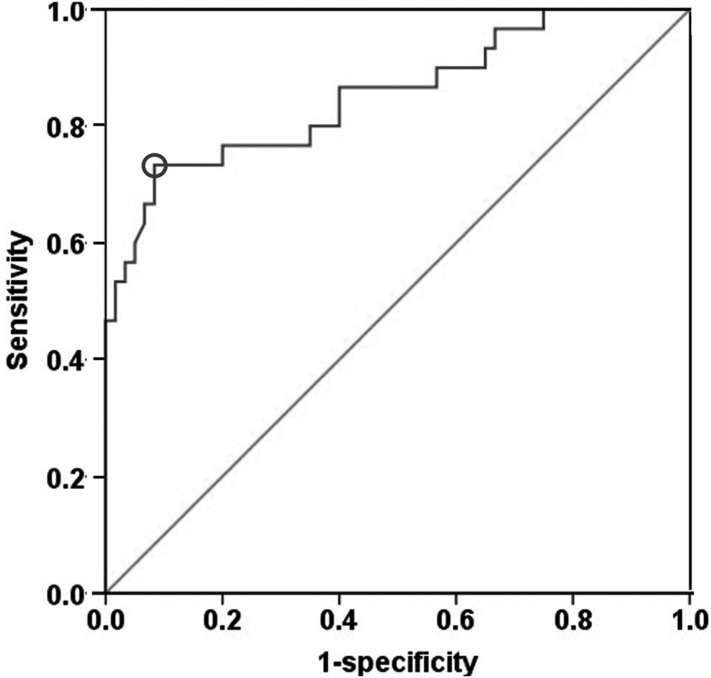
ROC curve analysis PSP/reg predicts the incidence of DKD. The 30.4 ng/mL cut-off reveals a sensitivity of 73.3 % and a specificity of 91.7 % to predict the incidence of DKD.

We set out to assess the predictive value of PSP/reg in T2DM by receiver operating characteristic (ROC) analysis. The area under the curve (AUC) of PSP/reg for presence of DKD was 0.854 (Figure 3). Furthermore, ROC analysis revealed that the optimal cut-off point of PSP/reg was 30.4 ng/ml to indicate DKD (Youden index = 0.65, sensitivity, 73.3%; specificity, 91.7%).

## DISCUSSION

DKD is one of the most prevalent chronic complications in patients with diabetes. Glomerular injury is regarded as an early sign of DKD, and microalbuminuria is a potent indication of DKD progression [20]. Here we show that PSP/reg is significantly increased during progression of DKD, correlating with major parameters of T2DM.

Increasing observations suggest that many diabetic patients with normoalbuminuria might develop nephropathy [21]. On the other hand, not all patients with proteinuria are likely to develop progressive renal dysfunction [22, 23]. This demonstrates that different mechanisms might contribute to the pathogenesis of DKD. In recent years, a substantial body of evidence suggests that immune and inflammatory processes play a critical role in the development and progression of DKD. Several studies suggested that cytokines, such as tumor necrosis factor-α (TNF- α) and interleukin-6 (IL-6) [11, 24, 25], and chemokines, e.g. monocyte chemoattractant protein-1 (MCP-1), and C-X-C chemokine ligand 16 (CXCL16) [26], were involved in the inflammatory process of DKD.

In our previous study, elevated PSP/reg levels were found in patients with T2DM and diabetic chronic complications as compared to healthy controls [19]. In the present study, we analyzed PSP/reg levels in patients with DKD and revealed a close relationship between PSP/reg levels and DKD in T2DM patients. Serum PSP/reg was significantly increased in the DKD group as compared to T2DM without DKD patients and more importantly, high serum PSP/reg level seemed associated with renal injury and insufficiency. These results further imply that inflammation may be important in facilitating the development and progression of DKD [4].

In the current investigation, we found correlations between PSP/reg levels and HbA1c, as well as fasting blood glucose(FBG) and 2-h postprandial glucose(2hPG), implying that the elevation of PSP/reg levels might have a close association with beta cell dysfunction. This finding is in accordance with recent data suggesting that upregulated extracellular glucose concentrations reinforce gene expression of PSP/reg, which may indicate an important feedback loop for the regulation of beta-cell mass [27]. Meanwhile, consistent positive correlations of PSP/reg levels with serum creatinine, UA and negative correlation with eGFR suggest that serum PSP/reg levels closely reflect glomerular injury and declining renal function in T2DM patients.

For future risk assessment, we calculated the AUC of PSP/reg for presence of DKD, which was 0.854 (95 %CI: 0.764-0.944). In the process of this analysis, we identified a cut-off value for PSP/reg at 30.4 ng/ml in T2DM patients. This was the most significant parameter associated with the occurrence of DKD. In our previous study, a PSP/reg cut-off of 22 ng/ml in nondiabetic group was found to indicate the incidence of T2DM. Here, we investigated this higher cut-off to have a better predictive value for DKD.

In summary, our study provides clinical evidence that serum levels of PSP/reg are highly increased in subjects with DKD and are positively associated with HbA1c and negatively with eGFR. These data imply that PSP/reg may be associated with renal injury in T2DM patients. There are also some limitations in our study. First, as a preliminary study, the sample size of this study is relatively small, and as the nature of a cross-sectional design, further prospective studies with larger study population and different stages of DKD patients are required to determine whether PSP/reg can be used as a potential biomarker for diagnosing and evaluating the onset and development of DKD. Second, to strengthen our results on the role of PSP/reg as a marker of renal injury and inflammation in the disease progression of DKD, correlations with serum cystatin C (Cys C), homocysteine (Hcy) and other inflammatory markers such as C reactive protein (CRP), tumor necrosis factor-alpha (TNF-α) are needed.

Despite these limitations, our preliminary data suggest that PSP/reg may help detect patients at high risk for kidney injury in T2DM. Therefore, it is worth considering the potential role of serum PSP/reg level as a supplementary indicator of DKD. An enhanced understanding of the inflammatory response in the diabetic kidney may be beneficial to facilitate the identification of novel therapeutic strategies for the treatment of DKD.

## MATERIALS AND METHODS

### Ethics

The study was approved by the institutional clinical research ethics committee of Zhongda Hospital, Southeast University and adhered to the principles of the Declaration of Helsinki. All subjects provided informed consent to participate in the study.

### Study population

A total of 120 subjects including those of newly diagnosed T2DM patients (*n* = 30), diabetes patients without DKD (*n* = 30) and with DKD (*n* = 30), as well as healthy controls (*n* = 30) were enrolled from Zhongda Hospital, Southeast University, China. The diagnostic criteria for T2DM was based on American Diabetes Association (ADA) criteria 2014. DKD was defined according to either the presence of microalbuminuria (30 to 299 mg albumin/24 h or an albumin to creatinine ratio [ACR] of 30 to 299 mg/g) or macroalbuminuria (> 300 mg albumin/24 h or ACR > 300 mg/g). DKD was also classified on the basis of renal biopsy.

Subjects with any of the following conditions were excluded from this study:(1) T1DM; (2) acute complications of diabetes including diabetic hyperosmolar coma, ketoacidosis, lactic acidosis, hypoglycemic coma; (3) history of other renal disease except DKD; (4) active or chronic infection or inflammatory disorders, neoplastic disorders, severe liver dysfunction; (5) pregnancy; (6) on medicine which affects blood or urine glucose levels.

We also included 30 healthy volunteers who underwent a regular health examination at Zhongda Hospital, Southeast University, China. All healthy subjects were selected based on the results of laboratory tests and a physician's questionnaire.

### Data collection

A full clinical assessment was obtained by a standardized questionnaire. Anthropometric measurements including height, weight, waistline, hipline, and waist hip rate (WHR) were obtained. BMI was calculated as weight (kg) divided by the square of the height (m). Personal information such as age, gender, smoking behavior, hypertension, duration of diabetes, family history of diabetes, medical history was also recorded. All participants underwent an oral glucose tolerance test (OGTT) after a 12-h overnight fast with measurement of FBG, 2hPG, and HbA1c. Blood samples were collected in BD vacutainers, and then centrifuged at 1500xg for 20 minutes. Serum was aliquoted and frozen at −80°C until the assay was performed. Other clinical biochemical parameters, such as total cholesterol (TC), triglyceride (TG), high-density lipoprotein (HDL), low-density lipoprotein (LDL); HbA1c, creatinine (Cr), blood urea nitrogen (BUN), eGFR, CCr, UA were measured based on standard methods.

### PSP/reg enzyme-linked immunosorbent assay

The ELISA to quantify human PSP/reg was performed as previously described [15], using Guinea pig anti-human recombinant PSP/reg antibodies. Deep frozen serum samples were used for PSP/reg measurements, and the IgG were purified by affinity chromatography on protein A columns. A sandwich ELISA was performed on 96-well plates. The plates were blocked with 1% bovine serum albumin (BSA) in Tris-buffered saline (TBS) for 1 h. Guinea pig anti-PSP/reg antibody was then coated to the bottom. Subsequently, samples were pre-diluted in TBS/BSA and loaded in duplicate wells, incubating for 2 h. After washing, a second antibody, rabbit anti-PSP/reg, was then incubated and detected by phosphatase-coupled anti-rabbit IgG [28]. The reaction of the phosphatase with a substrate was determined on a multiplate reader (Dynatech), and subjects' serum PSP/reg levels were compared with standard amounts of recombinant human PSP/reg protein.

### Statistical analysis

Statistical analyses were performed by the SPSS 19.0 software. Data are shown as mean ± standard deviation (SD) or as percentages for normal distribution. Non-normally distributed values are presented as median (interquartile range [IQR]). Correlations of PSP/reg and clinical parameters were performed using Pearson's correlations or Spearman's rank correlation coefficient when appropriate. Differences of PSP/reg between groups were determined by analysis of variance (ANOVA). AUC values were presented with 95 % confidence intervals (95%CI). Cut-off was identified by Youden's index. Correlations and differences were defined as significant at *p* < 0.05.

## SUPPLEMENTARY MATERIALS FIGURES AND TABLES


