# Supplementary material for: Elevated serum level of pancreatic stone protein/regenerating protein (PSP/reg) is observed in diabetic kidney disease
Source: Oncotarget. 2017 Mar 18;8(24):38145–51. doi: 10.18632/oncotarget.16369 (PMC5503521; doi:10.18632/oncotarget.16369)
Supplement: Supplementary file 1 [file oncotarget-08-38145-s001.pdf]

Elevated serum level of pancreatic stone protein/regenerating protein (PSP/reg) is observed in diabetic kidney disease

Supplementary Material

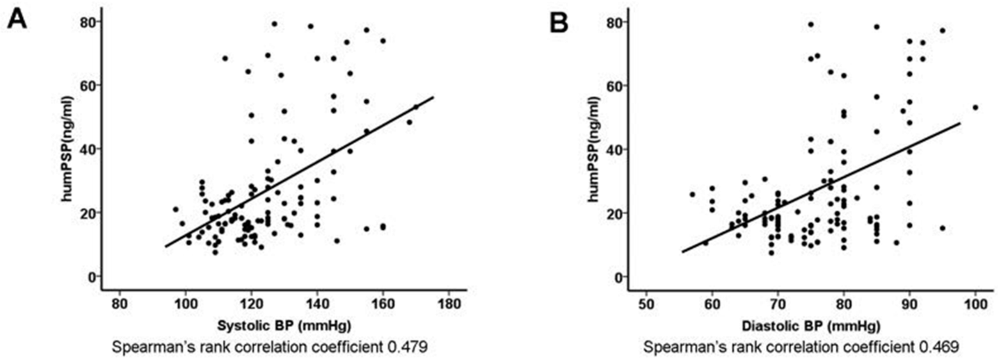

Supplementary Figure S1: Correlation analysis of PSP/reg and blood pressure. A. Correlation of PSP/reg with systolic blood pressure (Spearman r = 0.479, $p < 0.001$). B. Correlation of PSP/reg with diastolic blood pressure (Spearman r = 0.469, $p < 0.001$).
